# Severe *Streptococcus pyogenes* Infections, United Kingdom, 2003–2004

**DOI:** 10.3201/eid1402.070888

**Published:** 2008-02

**Authors:** Theresa L. Lamagni, Shona Neal, Catherine Keshishian, Neelam Alhaddad, Robert George, Georgia Duckworth, Jaana Vuopio-Varkila, Androulla Efstratiou

**Affiliations:** * Health Protection Agency, London, United Kingdom; † National Public Health Institute, Helsinki, Finland

**Keywords:** Streptococcus pyogenes, population surveillance, bacteremia, shock, septic, United Kingdom, research

## Abstract

As part of a Europe-wide initiative to explore current epidemiologic patterns of severe disease caused by *Streptococcus pyogenes*, the United Kingdom undertook enhanced population-based surveillance during 2003–2004. A total of 3,775 confirmed cases of severe *S. pyogenes* infection were identified over 2 years, 3.33/100,000 population, substantially more than previously estimated. Skin/soft tissue infections were the most common manifestation (42%), followed by respiratory tract infections (17%). Injection drug use was identified as a risk factor for 20% of case-patients. One in 5 infected case-patients died within 7 days of diagnosis; the highest mortality rate was for cases of necrotizing fasciitis (34%). Nonsteroidal antiinflammatory drugs, alcoholism, young age, and infection with *emm*/M3 types were independently associated with increased risk for streptococcal toxic shock syndrome. Understanding the pattern of these diseases and predictors of poor patient outcome will help with identification and assessment of the potential effect of targeted interventions.

## Abstract 2

Epidemiology of severe disease caused by this organism has changed, with increased incidence and different risk groups.

Diseases caused by the Lancefield group A streptococcus *Streptococcus pyogenes* are among the most varied in terms of clinical spectra and severity, ranging from the ubiquitous pharyngitis to rarer life-threatening manifestations such as necrotizing fasciitis. Interest in these diseases was renewed after the United States and several countries in Europe reported increasing numbers of cases of invasive *S. pyogenes* disease during and since the 1980s (*1*). These apparent changes triggered several rapid global initiatives, coordinated by a World Health Organization working group, including review of microbiologic diagnostic methods and commencement of enhanced surveillance in several countries during the mid-1990s (*2*).

In light of these changes, a cohesive network of 11 countries was formed in September 2002 to gain insight into the epidemiology of severe *S. pyogenes* disease across Europe. This network was funded by the European Union Fifth Framework Programme (*3*). To meet the Strep-EURO program objectives, the United Kingdom and other countries established population-based enhanced surveillance of severe *S*. *pyogenes* disease. Surveillance was undertaken to obtain accurate and comparable measures of overall and disease-specific incidence among participants and to compare demographic, risk factor, and clinical profiles of case-patients between countries, as well as microbiologic characteristics of *S*. *pyogenes* isolates collected.

## Methods

In accordance with the program objectives, the Public Health Laboratory Service (now part of the Health Protection Agency) initiated enhanced surveillance of severe *S. pyogenes* disease from January 1, 2003, through December 31, 2004. Cases were defined according to the US definition (*S*. *pyogenes* isolated from a sterile site) (*4*). Also included were cases in which *S*. *pyogenes* was isolated from a nonsterile site in combination with probable streptococcal toxic shock syndrome (STSS) or another severe manifestation (pneumonia, necrotizing fasciitis, puerperal sepsis, meningitis, or septic arthritis). STSS was defined according to US specifications that differentiate between confirmed and probable cases on the basis of recovery of a sterile or nonsterile site isolate, respectively (*4*).

To maximize case ascertainment, cases were identified from 2 sources: isolate referrals to the national reference laboratory (Streptococcus and Diphtheria Reference Unit [SDRU]) and surveillance reports made to the Communicable Disease Surveillance Centre (CDSC) (*5*). SDRU provides reference microbiology services to the United Kingdom; CDSC surveillance covers England, Wales, Northern Ireland, the Channel Islands, and the Isle of Man. Cases identified from each source were reconciled with each other by using automated techniques to match records on the basis of personal identifiers (date of birth, sex, hospital number, National Health Service number, SOUNDEX-coded surname) and geographic location, followed by loose matching and manual checking to allow records to differ slightly on any given matching parameter. Referring laboratories were sent a study questionnaire to obtain further information on the demographic profile of the patient, disease manifestations, markers of clinical severity, outcome, and possible sources of infection (*6*). Ethnicity of patients was sought and classified according to census groupings for rate calculation. Where an isolate had not been received by SDRU, this isolate was also requested.

Group A streptococcal isolates referred to SDRU were characterized according to their M protein by using conventional serologic and *emm* gene typing (*7**,**8*). Antimicrobial drug–susceptibility testing was conducted by referral laboratories according to local standard operating procedures.

Responses to completed questionnaires were entered and stored in a custom-made Access (Microsoft, Redmond, WA, USA) database. All reports were checked to ensure they met the case definition. Repeat episodes were defined as those occurring in the same patient >30 days after the initial episode; reports received within 30 days were considered part of the same episode. Data were extracted for statistical analysis into STATA statistical software release 8.2 (Stata Corporation, College Station, TX, USA). Descriptive statistics were undertaken on confirmed cases with χ^2^ and *t* tests used to test statistical significance of differences between subgroups. Incidence rates were calculated by using midyear resident population estimates for the respective years, age groups, sexes, and regional populations, with exact 95% confidence intervals (CIs) calculated according to the Poisson distribution. The 2001 census data were used as denominators for calculating rates according to ethnic group. All denominators were obtained from the Office for National Statistics. Stepwise unconditional logistic regression analysis was conducted to examine the independence of explanatory variables and development of STSS; the likelihood ratio test was used to evaluate significance of explanatory variables within each model.

All analyses were made on data from the United Kingdom, Channel Islands, and Isle of Man, except for estimated rates of infection, which were calculated for the areas with dual reporting (England, Wales, Northern Ireland, Channel Islands, and Isle of Man). The last 2 areas were omitted for age-, sex-, and ethnicity-specific rate calculations because of unavailability of these population denominators.

## Results

### Overview of Surveillance Results

From January 1, 2003, through December 31, 2004, a total of 3,821 cases of severe *S*. *pyogenes* disease meeting the case definition were reported from laboratories across the United Kingdom, Channel Islands, and Isle of Man. Of these cases, 21% were identified from isolate referrals only, without a corresponding surveillance report. Among the 3,821 reports were 46 repeat episodes, 5 of which were third episodes. Excluding repeat episodes, severe *S*. *pyogenes* disease was diagnosed for 3,775 patients in the United Kingdom, Channel Islands, and Isle of Man in 2003 and 2004.

*S*. *pyogenes* was isolated from a sterile site from 3,742 (99%) case-patients, primarily from blood culture (89%, 3,352). Thirty-three cases without sterile site isolates were included on the basis of >1 of the following clinical indicators: probable toxic shock syndrome (13 cases), necrotizing fasciitis (15), pneumonia (4), and puerperal sepsis (3).

Questionnaires were received for 2,647 (70%) of 3,775 case-patients. Information available for case-patients for whom questionnaires were or were not returned indicated their similarity in terms of age (median age 48 and 45 years, respectively), sex (54% male for both), and strain characteristics (*emm*/M type and erythromycin resistance), although a slightly higher proportion of case-patients for whom a questionnaire was returned had disease onset in December–April (53% vs. 48%; χ^2^ 6.37, degrees of freedom [df] 1, p = 0.012).

### Geographic Distribution of Cases

In 2003 and 2004 combined, the overall rate of severe *S*. *pyogenes* infections was 3.33/100,000 population for England, Wales, Northern Ireland, Channel Islands, and Isle of Man. Variations were seen across these countries, with report rates higher in England (3,413 cases, 3.41/100,000) than Wales (153 cases, 2.60/100,000, rate ratio [RR] 1.32, 95% CI 1.12–1.55) or Northern Ireland (72 cases, 2.11/100,000, RR 1.62, 95% CI 1.28–2.04) but not higher than rates in the Channel Islands and Isle of Man (10 cases, 1.98/100,000, RR 1.55, 95% CI 0.83–2.88). Substantial variations were also apparent between the English regions, with rates higher in Yorkshire and Humber (4.92/100,000) than in any other English region: East Midlands (3.25), East of England (2.98), London (2.75), North East (3.66), North West (3.70), South East (2.79), South West (3.92), and West Midlands (3.51). Report rates decreased in 2004 (1,718 cases, 3.12/100,000, RR 0.89, 95% CI 0.83–0.95) compared with 2003 (1,930 cases, 3.53/100,000); decreases in Yorkshire and Humber, and London accounted for 85% of this decrease.

### Demographic Profile of Case-Patients

**Figure 1:**
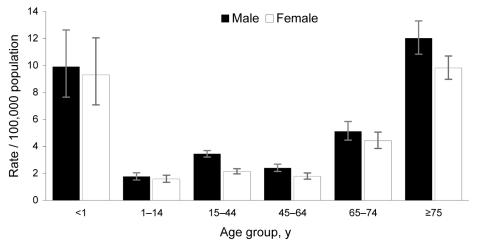
Age- and sex-specific annual incidence of severe *Streptococcus pyogenes* infection in England, Wales, and Northern Ireland, 2003–2004. Error bars show 95% confidence intervals.

Severe *S*. *pyogenes* infection reports were highly concentrated in elderly persons (>75 years of age, 10.67/100,000) and the young (<1 years of age, 9.70/100,000) (Figure 1). Rates for male patients were 22% higher than for female patients (3.65/100,000 vs. 2.98/100,000, RR 1.22; 95% CI 1.14–1.30); more male case-patients were found across all age groups, in particular, young adults (15–44 years of age), for whom rates were 61% higher for male than female patients (3.44/100,000 vs. 2.14/100,000, RR 1.61, 95% CI 1.43–1.80). Of 1,822 case-patients whose ethnicity was recorded, 1,727 (95%) were white, 58 (3%) from the Indian subcontinent, and 21 (1%) black African or Caribbean. Rates of severe *S. pyogenes* disease observed were significantly higher for whites (3.29) than for those of Indian subcontinent (2.46/100,000, RR 1.34, 95% CI 1.02–1.74) or black African or Caribbean origin (1.84/100,000, RR 1.79, 95% CI 1.16–2.75).

### Seasonal Patterns of Infection

**Figure 2:**
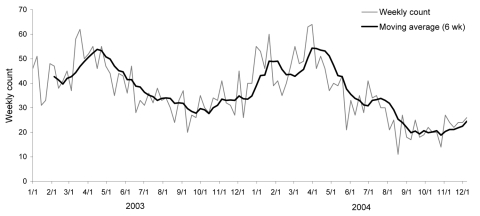
Seasonal trends in reports of severe *Streptococcus pyogenes* infection in the United Kingdom, 2003–2004. Moving average (6 wk) is the average count for the previous 6 weeks.

Marked seasonal peaks of severe *S*. *pyogenes* infection were observed in both years. Cases gradually increased from the end of October and first peaked near the end of January (2nd week of 2003, 51 cases; 4th week of 2004, 60 cases) before peaking again (higher) toward the end of March (12th week of 2003, 62 cases; 14th week of 2004, 64 cases) (Figure 2).

### Clinical Manifestations of Cases

Clinical information was reported on the study questionnaire for 2,611 (69%) severe cases of *S*. *pyogenes* infection (Table 1). Approximately one fifth of patients were bacteremic but had no defined focus for infection. Of the remainder, skin/soft tissue was the most common focus of infection (42%, 1,099). Cellulitis was the most common manifestation, diagnosed for 881 (34%) patients; necrotizing fasciitis was diagnosed for 136 (5%) patients. Necrotizing fasciitis was rarely reported for pediatric case-patients (<16 years of age, 1%); most (64%, 87/136) cases occurred in persons 16–60 years of age. The respiratory tract was the next most commonly affected system; 434 (17%) patients showed clinical signs of upper or lower respiratory tract infection. Pneumonia was diagnosed for 309 (12%) case-patients.

**Table 1:** Clinical manifestations of severe *Streptococcus pyogenes* infection, United Kingdom, 2003–2004

Manifestation	All case-patients, no. (%)*	Children <16 y of age, no. (%)	Adults 16–60 y of age, no. (%)	Adults >60 y of age, no. (%)
Skin/soft tissue infection	1,099 (42)	85 (27)	479 (39)	531 (50)
Cellulitis	881 (34)	59 (19)	362 (30)	457 (43)
Necrotizing fasciitis	136 (5)	4 (1)	87 (7)	45 (4)
Abscess	134 (5)	6 (2)	112 (9)	16 (2)
Erysipelas	24 (1)	2 (1)	9 (1)	13 (1)
Bacteremia with no defined focus	558 (21)	77 (24)	228 (19)	252 (24)
Respiratory tract infection	434 (17)	66 (21)	187 (15)	181 (17)
Pneumonia	309 (12)	27 (8)	139 (11)	143 (13)
Other lower respiratory tract infection	62 (2)	6 (2)	26 (2)	30 (3)
Pharyngitis/tonsillitis	51 (2)	26 (8)	16 (1)	9 (1)
Ear infection	22 (1)	8 (3)	9 (1)	5 (<1)
Epiglottitis	17 (1)	2 (1)	10 (1)	5 (<1)
Sinusitis	6 (<1)	4 (1)	0	2 (<1)
Septic arthritis	220 (8)	40 (13)	114 (9)	66 (6)
Puerperal sepsis	58 (2)	0	58 (5)	0
Acute abdominal infection†	49 (2)	5 (2)	31 (3)	12 (1)
Cardiac infection‡	48 (2)	2 (1)	36 (3)	10 (1)
Meningitis	37 (1)	18 (6)	12 (1)	7 (1)
Total	2,611 (100)	318 (100)	1,225 (100)	1,064 (100)

*Includes patients without age information. †Includes 14 patients with peritonitis, 1 with appendicitis, and 1 with pancreatitis. ‡Includes 42 patients with endocarditis, 4 with pericarditis, 1 with myocarditis, 1 with myocarditis and pericarditis.

Confirmed STSS developed in 196 (8%) patients who had a diagnosis of severe *S*. *pyogenes* infection (Table 2); 28% of these patients had necrotizing fasciitis. STSS developed in 40% (55/136) of patients with necrotizing fasciitis compared with 6% of other patients (χ^2^ 224.14, df 1, p<0.001). Use of nonsteroidal antiinflammatory drugs was positively associated with development of STSS (22% vs. 8%, χ^2^ 13.71, df 1, p<0.001).

**Table 2:** Clinical markers of severe *Streptococcus pyogenes* infection in the United Kingdom, 2003–2004*

Marker	All case-patients (n = 2,611), no. (%)	Bacteremia with no defined focus (n = 558), no. (%)	Pneumonia (n = 309), no. (%)	Cellulitis (n = 881), no. (%)	Septic arthritis (n = 220), no. (%)	Necrotizing fasciitis (n = 136), no. (%)	Puerperal sepsis (n = 58), no. (%)
Clinical severity (n = 2,611)							
Hypotensive shock	494 (19)	101 (18)	70 (23)	155 (18)	43 (20)	80 (59)	13 (22)
STSS (hypotension + >2 listed below)	196 (8)	28 (5)	27 (9)	63 (7)	18 (8)	55 (40)	3 (5)
Renal impairment	381 (15)	69 (12)	50 (16)	149 (17)	40 (18)	58 (43)	3 (5)
Respiratory distress	288 (11)	41 (7)	113 (37)	67 (8)	20 (9)	33 (24)	1 (2)
Erythematous rash	231 (9)	30 (5)	15 (5)	135 (15)	13 (6)	17 (13)	4 (7)
Soft-tissue necrosis	254 (10)	0	8 (3)	116 (13)	13 (6)	136 (100)	3 (5)
Liver abnormality	185 (7)	31 (6)	23 (7)	65 (7)	14 (6)	31 (23)	3 (5)
Disseminated intravascular coagulation	131 (5)	21 (4)	14 (5)	40 (5)	10 (5)	35 (26)	6 (10)
Admission to ICU (n = 2,292)	451 (20)	76 (16)	68 (24)	136 (17)	40 (21)	101 (77)	8 (16)
Surgical intervention (n = 1,885)	443 (24)	21 (6)	19 (8)	131 (20)	106 (63)	106 (87)	10 (24)
Death within 7 d (n = 2,192)	413 (19)	97 (22)	87 (32)	121 (16)	17 (9)	43 (34)	2 (4)

*STSS, streptococcal toxic shock syndrome; ICU, intensive care unit.

Multivariable analysis of patient, clinical, and microbiologic factors associated with development of STSS identified age to be a strong predictor for STSS; risk for STSS was 5-fold greater for persons 15–44 years of age (odds ratio [OR] 5.42, 95% CI 2.22–13.23, p<0.001) than for the reference group (children <15 years of age). Persons 45–64 years of age had a 5-fold increased risk for STSS (OR 5.20, 95% CI 2.12–12.74, p<0.001). Patients >65 years of age had no increased risk for STSS compared with the pediatric reference group. Regardless of age, patients with necrotizing fasciitis had a 7-fold increased risk for STSS (OR 6.87, 95% CI 4.25–11.09, p<0.001).

Four risk factors (alcoholism, injection drug use, malignancy, and use of nonsteroidal antiinflammatory drugs) were independently associated with development of STSS. Patients who used nonsteroidal antiinflammatory drugs had a 3-fold increased risk for STSS (OR 3.00, 95% CI 1.30–6.93, p = 0.01). Alcoholism was associated with a 2-fold increased risk for STSS (OR 2.52, 95% CI 1.27–5.03, p = 0.008). Conversely, patients with malignancies had a much lower risk for STSS (OR 0.34, 95% CI 0.12–0.96, p = 0.042), as did injection drug users (OR 0.23, 95% CI 0.10–0.56, p = 0.001). Patients infected with an *emm*/M3 type, which was the only strain associated with STSS, had a 3-fold increased risk for STSS compared with patients infected with the reference group (*emm*/R28) (OR 3.20, 95% CI 1.35–7.58, p = 0.008).

Overall, 413 (19%) patients for whom *S*. *pyogenes* infection was the main underlying or contributory cause of death died within 7 days of initial microbiologic diagnosis. Necrotizing fasciitis was the most severe clinical manifestations for patients, according to specified markers; patients with this condition were most likely to be admitted to an intensive care unit (ICU) (77%) or to die within 7 days of diagnosis (34%). However, ICU admission (20%) and surgical intervention (24%) were not uncommon among other patients. Case-fatality rates were also high for patients with pneumonia, 32% of whom died within 7 days. Of the 58 young women in whom puerperal sepsis developed, 2 died. Development of STSS was strongly linked to risk for death; 84 (45%) of 185 patients with STSS died from their infection compared with 329 (16%) of 2,007 without STSS.

### Patient Risk Factors

Information on risk factors was available for 61% of case-patients with severe *S*. *pyogenes* infection. Of these, lesions or wounds to the skin were reported for 31% (719). Skin is the most likely portal of entry recorded overall, especially among persons >60 years of age, 40% of whom had skin lesions. Information on the nature of these lesions was available for 617 case-patients. The 2 most common types were traumatic lesions (188) and chronic wounds (161). Traumatic lesions were most common among young adults (16–60 years of age, 9%); chronic wounds were most common among elderly persons (14%). Less common types of wounds were recorded that included insect bites (21 cases, 0.9%) and animal-associated traumatic wounds (cat scratches and dog and human bites, 8 cases, 0.3%).

Of young adults with severe *S*. *pyogenes* infections, 459 (40%) were injection drug users (20% of case-patients of all ages). Other conditions commonly reported that could have predisposed persons to infection included malignancies (161) and diabetes (158), each noted for 7% of cases overall and 11% and 13%, respectively, among elderly persons. Nine percent (204) of infections were associated with healthcare, mostly postsurgical infections (118). Among pediatric case-patients (<16 years of age), varicella was the next most common predisposing factor noted after skin lesions, reported for 41 (14%) children. Overall, 566 (25%) case-patients did not have any particular predisposition, or risk for severe *S*. *pyogenes* infection on the basis of the common factors outlined (Table 3) or any others considered pertinent by the reporting clinician. Among pediatric case-patients, this proportion increased to 46% (132).

**Table 3:** Potential predisposing factors for severe *Streptococcus pyogenes* infection, United Kingdom, 2003–2004

Factor	All case-patients, no (%)*	Children <16 y of age, no. (%)	Adults 16–60 y of age, no. (%)	Adults >60 y of age, no. (%)
Skin lesion/wound	719 (31)	63 (22)	298 (26)	358 (40)
Trauma	188 (8)	13 (5)	99 (9)	76 (9)
Chronic wound	161 (7)	0	39 (3)	122 (14)
Surgery	118 (5)	10 (4)	48 (4)	60 (7)
Injection drug use	459 (20)	0	459 (40)	0
Healthcare-associated infection	204 (9)	15 (5)	79 (7)	109 (12)
Malignancy	161 (7)	11 (4)	49 (4)	100 (11)
Diabetes	158 (7)	3 (1)	40 (4)	115 (13)
Alcoholism	88 (4)	0	67 (6)	21 (2)
Recent childbirth	86 (4)	0	86 (8)	0
Steroid use	77 (3)	3 (1)	35 (3)	39 (4)
Contact with person with *S. pyogenes* infection†	57 (4)	7 (4)	34 (5)	16 (2)
Nonsteroidal antiinflammatory drug use	49 (2)	3 (1)	18 (2)	28 (3)
Varicella	47 (2)	41 (14)	4 (<1)	2 (<1)
Cardiovascular disease	45 (2)	3 (1)	9 (1)	33 (4)
Upper respiratory tract infection	39 (2)	9 (3)	24 (2)	6 (1)
Renal impairment	31 (1)	2 (1)	19 (2)	10 (1)
Other reported risk factor‡	112 (5)	30 (11)	41(4)	41 (5)
No risk factors reported	566 (25)	132 (46)	173 (15)	260 (29)
Total	2,305 (100)	284 (100)	1,135 (100)	884 (100)

*Includes patients without age information. †Information available for 1,586 patients. ‡Noted for <30 patients.

## Discussion

As part of a wider European initiative to improve our understanding of the epidemiology of severe *S*. *pyogenes* infections, the United Kingdom has amassed one of the largest collections of such cases recorded. The >3,700 cases diagnosed in 2003–2004 resulted in a rate of 3.33/100,000 population for England, Wales, Northern Ireland, the Channel Islands, and Isle of Man. This rate was similar to rates reported for other European countries and the United States in the early 2000s (*1*,*9*,*10*), although lower than some estimates from Canada (*11*–*13*). In the 2-year study period, the overall rate of severe *S*. *pyogenes* infections decreased from 3.53/100,000 to 3.12/100,000. This overall decrease was largely caused by a substantial decrease in 2 regions, Yorkshire and Humber, and London. Use of multiple sources for case ascertainment was an improvement over previously used methods. Previous methods, which relied solely on voluntary laboratory reporting, would have yielded a rate of 2.65/100,000 during this period. As with any study dependent on participation of local reporters, this study may have missed additional diagnosed cases.

Rates of severe diseases associated with *S*. *pyogenes* were markedly higher in male patients than in female patients, an observation not consistently found in other countries (*12*,*14*) but commonly found among patients with bacteremic infections in the United Kingdom (*15*,*16*). We did not observe any increased rate of severe *S*. *pyogenes* infections in black patients of African or Caribbean origin, as was found in a large study in the United States (*17*). Estimated rates were lower for black patients than for Asian or white patients. Because our study relied on clinician and microbiologist reporting of ethnicity, the proportion of patients reported as white may have been overestimated as a result of assumptions made without confirmatory information.

Marked seasonal patterns in severe *S*. *pyogenes* disease were evident during the study period, with an initial peak in December–January, followed by a strong peak in March–April. Preliminary comparisons among Strep-EURO participants suggest similar patterns in other European countries (*18*). Why these diseases should peak in late winter and early spring is not known. Seasonal patterns of viral respiratory infections with respiratory syncytial virus and influenza virus, which could make patients vulnerable to invasive *S*. *pyogenes* infections, may play a role in early- to mid-winter *S*. *pyogenes* peaks but would not explain the main spring peak seen in the United Kingdom (Health Protection Agency, unpub. data).

Clinical information provided for these case-patients highlights the severity of these infections; 19% died within 7 days of the initial culture-positive specimen being obtained. This finding is consistent with overall case-fatality estimates during enhanced surveillance in the United Kingdom in 1994–1997 (25%) and estimates reported in other countries (*14*,*17*,*19*,*20*). However, only 1 of these studies defined a time frame for estimates or included the role of infection in the patient’s death, as our study did. Case-fatality rates were particularly high for case-patients with necrotizing fasciitis, who accounted for only 5% of all cases but 10% of all deaths. Completion of the questionnaire could also have been biased in favor of more severe or interesting cases.

Among case-patients identified in this study, 12% had pneumonia, a value substantially higher than that noted by enhanced surveillance in the United Kingdom in 1994–1997 (5%) (*20*). However, our finding was consistent with those in studies in other countries (*11*,*13*,*17*,*19*,*21*,*22*). The case-fatality rate in our study (32% within 7 days of initial diagnosis) was substantially higher than that expected with community-acquired pneumonia (*23*).

STSS developed in 8% of the case-patients identified in our study. These patients had poor survival rates; 45% died within a week of initial diagnosis. STSS was most likely to develop in young adults, which is consistent with findings of a US study that reported a lower median age for STSS patients (*24*). Infection with an *emm*/M3 strain was associated with an increased risk for STSS. This finding is consistent with previous (unadjusted) findings from the United States (*17*) but different from findings from Canada, which only identified *emm*/M9, a strain uncommon in the United Kingdom, as associated with STSS (*13*). Alcoholism was associated with increased risk for STSS; this association was also found in other studies (*13*,*22*). However, risk for STSS was 3-fold greater for patients who were reported to have used nonsteroidal antiinflammatory drugs, despite adjustment for whether patients had necrotizing fasciitis, which is strongly associated with STSS. Because no data were collected about time, dose, indications for use, or which agent was taken, a causal link between use of nonsteroidal antiinflammatory drugs and STSS cannot be inferred from our findings. A confounding factor, such as delay in receiving appropriate treatment, which we did not adjust for in our analysis, could explain this finding. Patients who took nonsteroidal antiinflammatory drugs may have had early signs, such as extreme pain, which indicated a more severe infection. Nonetheless, this remains an interesting and potentially important observation in a controversial area where evidence supporting either immunologic impairment or augmentation caused by nonsteroidal antiinflammatory drugs remains unresolved (*25*). Other novel associations with STSS were made; e.g., STSS was less likely to develop in injection drug users or patients with malignancies than in other patients. These findings point to immunocompetence as a necessary mediator for development of STSS.

Questionnaires concerning possible predisposing factors have highlighted skin lesions as the most commonly identified potential source of infection, which is similar to findings in other countries (*18*,*22*). However, given the occult nature of *S*. *pyogenes* infections, as indicated by the high proportion (21%) of cases with no identified focus, several of these cases may have originated from respiratory tract colonization. This colonization could lead to transient bacteremia, which in turn seeded local tissue sites, possibly in the presence of local trauma.

In contrast to preliminary findings from other Strep-EURO participants (*18*), a substantial proportion of case-patients were injection drug users (20% overall and 40% among young adults). Regional breakdowns of risk factors were not undertaken in our analyses. However, regional differences in report rates are in part explainable by injection drug use–related cases, with highest overall rates in the Yorkshire and Humber region. This region had been identified as having a particular problem of severe *S*. *pyogenes* disease in injection drug users (*26*). Patterns of isolate referrals to the national reference laboratory over the past decade suggest an increase in severe *S*. *pyogenes* infection in injection drug users (*27*). The reasons behind this change remain unclear and warrant further investigation.

Most severe *S*. *pyogenes* cases in our study occurred sporadically in the community; only 9% were associated with healthcare interventions. One fourth of all case-patients and nearly 1 in 6 young adults had no particular risk factors identified. These findings highlight the likely economic effect of these infections and the challenges in developing any effective prevention measure.

Analysis of information collected in this study has yielded some unique insights into these infections and has begun to provide an evidence base for mounting public health initiatives in the United Kingdom (*28*). High and rapidly ensuing mortality rates among these patients emphasize the need for early recognition and rapid treatment. Maintaining a high index of suspicion, especially where there are signs of possible necrotizing fasciitis, could clearly be life-saving. Further analysis of *emm*/M type distribution, a key virulence factor inducing immunologic memory, will assist in assessment of the potential effect of vaccines currently under development. Changes in the epidemiology of severe *S*. *pyogenes* infections since the last period of enhanced surveillance in the mid-1990s underline the need for periodic monitoring to detect changes in disease manifestations, risk groups, and microbiologic characteristics to develop strategies for control and management of these infections.
